# The influence of different concentrations of bio-organic fertilizer on cucumber Fusarium wilt and soil microflora alterations

**DOI:** 10.1371/journal.pone.0171490

**Published:** 2017-02-06

**Authors:** Nan Huang, Weiwei Wang, Yanlai Yao, Fengxiang Zhu, Weiping Wang, Xiaojuan Chang

**Affiliations:** 1 The College of Life Sciences, Northwest University, Xi’an, Shaanxi, China; 2 Key Laboratory of Resource Biology and Biotechnology in Western China, College of Life Sciences, Northwest University, Ministry of Education, Xi’an, Shaanxi, China; 3 Institute of Environment, Resource, Soil and Fertilizer, Zhejiang Academy of Agricultural Sciences, Hangzhou, Zhejiang, China; Universita degli Studi di Pisa, ITALY

## Abstract

Fusarium wilt is one of the main diseases of cucumber, and bio-organic fertilizer has been used to control Fusarium wilt. In this study, a pot experiment was conducted to evaluate the effects of bio-organic fertilizer applied at four levels on the suppression of Fusarium wilt disease in cucumber, the soil physico-chemical properties and the microbial communities. In comparison with the control (CK), low concentrations of bio-organic fertilizer (BIO2.5 and BIO5) did not effectively reduce the disease incidence and had little effect on soil microorganisms. High concentrations of bio-organic fertilizer (BIO10 and BIO20) significantly reduced the disease incidence by 33.3%-66.7% and the production was significantly improved by 83.8%-100.3%. The soil population of *F*. *oxysporum* f. sp. cucumerinum was significantly lower in bio-organic fertilizer treatments, especially in BIO10 and BIO20. The microorganism activity increased with the bio-organic fertilizer concentration. High-throughput sequencing demonstrated that, at the order level, *Sphingomonadales*, *Bacillales*, *Solibacterales* and *Xylariales* were significantly abundant in BIO10 and BIO20 soils. At the genus level, the abundance and composition of bacterial and fungal communities in BIO10 and BIO20 were similar, illustrating that high concentrations of bio-organic fertilizer activated diverse groups of microorganisms. Redundancy analysis (RDA) showed that *Xanthomonadales*, *Sphingomonadales*, *Bacillales*, *Orbiliales*, *Sordariales*, and *Mucorales* occurred predominantly in the BIO10 and BIO20. These microorganisms were related to the organic matter, available potassium and available phosphorus contents. In conclusion, a high concentration of bio-organic fertilizer application suppressed the Fusarium wilt disease and increased cucumber production after continuous cropping might through improving soil chemical condition and manipulating the composition of soil microbial community.

## Introduction

Fusarium wilt of cucumber is a common soilborne disease observed all over the world that is caused by *Fusarium oxysporum* f. sp. cucumerinum, a typical soil-born pathogen. Chemical fungicides, crop rotation and soil fumigation are usually used to control Fusarium wilt. However, chemical fungicides might lead to environment pollution, have toxic effects on human health, and create imbalances in the microbial community [1], and the effectiveness of crop rotation is limited once a disease outbreak occurs [2]. Soil fumigation is an effective strategy but can destroy the natural structure of the soil [3]. Therefore, biological control represents a potentially attractive alternative disease management approach and may eventually replace some overused chemical pesticides in agriculture [4]. Compost can stimulate the proliferation of antagonists in the soil and thus suppress soil-borne pathogens. However, the application of only compost at insufficient rates has yielded inconsistent levels of disease control [5]. Effective colonization of the root system by plant growth-promoting rhizobacteria (PGPR) is crucial for the development of the beneficial effects of compost for the biocontrol of plant disease [6]. However, soil inoculation of PGPR without a suitable organic substrate will not result in stable biological control against soil-born pathogens because inoculated antagonists compete for nutrients with other native soil microbes to survive. Some reports have shown that the activity of strain *Bacillus amyloliquefaciens* SQR9 could be enhanced through the solid fermentation of manure organic fertilizer with *B*. *subtilis* SQR 9 [7]. The product was designated as bio-organic fertilizer (BIO).

The disease suppression afforded by these PGPR has been linked to the direct or indirect inhibition of pathogens. The direct inhibition of pathogens develops through competition or antibiosis, whereas indirect inhibition develops through stimulation of plant defense mechanisms or changes in the soil microbial community composition and structure [8, 9]. The crop production, soil ecosystem and nutrient cycle activity are, in part, also dependent upon the functional processes related to the soil microbial community composition and structure. Different inputs of PGPR agents can cause changes in soil bacterial and fungal densities by suppressing or promoting microbial growth and activity. Previous reports have demonstrated that the control efficiencies of BIO are directly related to the ability of SQR9 to control *F*. *oxysporum* f. sp. cucumerinum. However, the effects of different concentrations of BIO on the soil microbial community have not been well characterized [10]. Recently, community-level physiological profiles (CLPPs) and high-throughput sequencing (Illumina MiSeq sequencing) were employed to understand functional microbial activity and microbial diversity [11]. Additionally, real-time PCR was used to investigate the variations in populations of *F*. *oxysporum* f. sp. cucumerinum [12].

The aim of this study was to evaluate the direct suppression of *F*. *oxysporum* f. sp. cucumerinum by different concentrations of BIO, explore the potential impact of different concentrations of BIO on soil characteristics, and determine the alterations of soil microbial communities caused by different concentrations of BIO.

## Materials and methods

### Soil and BIO

We sampled the 0–20 cm soil layer that had been planted with cucumber continuously for three years with perennial chemical fertilizer application in a greenhouse at an agricultural cooperative in Jiaxing, Zhejiang Province, China (no specific permission was required for soil sampling in this location and the field in this study did not involve endangered or protected species). Fusarium wilt of cucumber occurred normally at a rate of 20–60% before sampling. The sampled soil was passed through a 2-mm mesh sieve before the experiment.

The BIO used in this study was provided by the Jiangyin Lianye Biological Technology Co. Ltd., Jiangsu, China. The BIO consisted of the second solid fermentation of organic fertilizer (cattle manure composts) with *Bacillus amyloliquefaciens* SQR 9. The composition of BIO was listed in Table 1.

**Table 1:** The composition of bio-organic fertilizer.

Name	Organic matter (g/kg)	Total Nitrogen (g/kg)	Total potassium (g/kg)	Total phosphorus; (g/kg)	The number of strain SQR9 (CFU/g)
**BIO**	284	25.8	14.9	42	>10^7^

### Pot experiments

To investigate the effects of different concentrations of BIO on the control of cucumber Fusarium wilt and soil microorganisms, pot experiments were performed with the addition of BIO at concentrations of 0.25%, 0.5%, 1.0% and 2.0% to soil, with the corresponding treatments designated as BIO2.5, BIO5, BIO10 and BIO20, respectively. Chemical fertilizer at a concentration of 0.09% was used in the control (CK). The total content of nitrogen, phosphate, and potassium in the chemical fertilizer was 45% with a component ratio of 15%:15%:15%. The chemical fertilizer and BIO were both used as the base fertilizer and thoroughly mixed with the soil before cucumber planting. To ensure thorough incorporation, we fully incorporated the soil and fertilizer in the plastic film before placing in the pot. Two cucumber plants were grown in each pot, each block had four pots, and each treatment had three blocks. The cucumber seed of “Yuanbifeng” was provided by Xiaxian Yuanfeng Vegetable Research Institute, Shanxi, China. The seed was not coated with seed coating agent. Cucumber seeds were initially sown in seedling plug trays until the seedlings had two true leaves and then transplanted to pots. These pots with a 20-cm diameter and 30-cm height were loaded with 5 kg of soil.

Fusarium wilt of cucumber was observed and recorded. The cucumber production was weighed and recorded during the process. The disease incidence was calculated as the percentage of infected plants in each treatment and was evaluated when the disease emerged (>20% of leaves wilted). Soil samples were collected after 60 days and were divided into three portions: for Biolog analysis; for analysis of soil characteristics after drying naturally; and for molecular analysis of the microbial community and the relative number of *F*. *oxysporumf*. sp. cucumerinum and *B*. *subtilis* SQR 9, stored at -80°C.

### Analysis of soil characteristics

The soil pH, electrical conductivity (EC), organic matter (OM), total nitrogen (TN), available phosphorus (AP) and available potassium (AK) were determined using naturally dried soil. Five grams of naturally dried soil was weighed and placed in a beaker with 25 mL of distilled water. After mixing, the soil was allowed to rest for 30 min, and the pH and EC were then determined using a pH and conductivity meter (Mp521 Lab pH/conductivity meter, Japan). The OM and TN were determined on an element analyzer (Vario ISOTOPE cube, Germany). The AP was determined using a spectrophotometer (TU-1810, Beijing, China), and the AK was determined using a flame spectrophotometer [13].

### Soil DNA extraction

Soil DNA was extracted using a PowerSoil^®^ DNA Isolation Kit following the manufacturer’s protocol (Mo Bio Laboratories Inc., Carlsbad, CA, USA).

### Relative quantification of *F*. *oxysporum* f. sp. cucumerinum and *B*. *subtilis* SQR9

Real-time PCR was applied to relative quantify the number of *F*. *oxysporum* f. sp. cucumerinum and *B*. *subtilis* SQR 9 in soil. Extracted soil DNA was used as the template. The 18S rDNA and 16S rDNA gene was used as the reference gene. All the primers involved in the experiment are listed in Table 2. Real-time PCR reactions were conducted in 96-well plates (Applied Biosystems 7500 real-time PCR detection system, Life Technologies, USA). The reaction volume was 20 μL, which consisted of 10 μL of Premix Ex Taq^™^ (2×) (Takara, Japan), 0.4 μL of ROX Reference Dye II (50×), 1 μL of each primer, 2 μL of template DNA, and 5.6 μL of ddH_2_O. The 18S rDNA and 16S rDNA temperature program was as follows: 95°C for 1 min, followed by 40 cycles of 95°C for 5 s, 55°C for 30 s, and 72°C for 30 s. Each reaction was run in triplicate. Ct values was automatically performed by the system. Relative quantification was analyzed based on the threshold cycle (Ct) values and the 2^-△△Ct^ method [14].

**Table 2:** Primers used in the real-time PCR.

Primers	Sequence (5’–3’)	Product size (bp)	Reference
**FocF3**	AAACGAGCCCGCTATTTGAG	244	[15]
**FocR7**	TATTTCCTCCACATTGCCATG		
**NS1**	GTAGTCATATGCTTGTCTC	349	[16]
**Fung**	ATTCCCCGTTACCCGTTG		
**SQR9F**	CATGAGATGGCGGGCTTT	375	[17]
**SQR9R**	CGCATCCTCCCTGTCTTTG		
**515F**	GTGCCAGCMGCCGCGGTAA	291	[18]
**806R**	GGACTACVSGGGTATCTAAT		

### Community-level physiological profiles (CLPPs)

Eco-microplates (Biolog Inc., Hayward, Calif.) were used to determine differences in the activity of the microbial communities among the CK, BIO2.5, BIO5, BIO10 and BIO20 treatments [19]. Five grams of a fresh soil sample was diluted in 45 mL of sterile saline water (0.85% NaCl w/v) and shaken for 60 min. Then, the solution was diluted in a 10-fold series. Finally, 150 μL of the diluted sample at 10^−3^ was inoculated in each well of a Biolog EcoPlate and incubated at 25°C for 7 days. Data were recorded at 590 nm and 760 nm every day [20,21].

### High-throughput sequencing-based microorganism community analysis

Changes in the microbial communities during the experiment, including bacteria and fungi, were analyzed using high-throughput sequencing. The concentration and purity of the extracted soil DNA was monitored on 1% agarose gels. According to the concentration, the DNA was diluted to 1 ng/μL using sterile water.

A fragment covering the V3 + V4 region of the 16S rDNA gene and a fragment covering the ITS1 region in the 18S rDNA gene were selected to construct bacterial and fungal community libraries, respectively, through tag high-throughput sequencing. The barcoded, broadly conserved primers 341F (5’-CCTACGGGNGGCWGCAG-3’) and 805R (5’-GACTACHVGGGTATCTAATCC-3’) amplify the V3 + V4 region in the 16S rDNA gene, and the BITS (5’-ACCTGCGGARGGATCA-3’) and B58S3 (5’-GAGATCCRTTGYTRAAAGTT-3’) primers amplify the ITS1 region in the 18S rDNA gene [22,23]. All PCR reactions were carried out in 30 μL reactions containing 15 μL of Phusion^®^ High-Fidelity PCR Master Mix (New England Biolabs), 0.2 μM forward and reverse primers, and approximately 10 ng of template DNA. Thermal cycling consisted of initial denaturation at 95°C for 3 min, followed by 25 or 30 cycles for 16S rDNA or 18S rDNA, respectively. Each cycle involved denaturation at 95°C for 30 s, annealing at 55°C for 30 s, elongation at 72°C for 30 s, and 16°C for 2 min. The same volume of 1*loading buffer (containing SYBR green) was mixed with the PCR products and submitted to electrophoresis on a 2% agarose gel for detection. Samples with a bright main stripe at 460 bp (V3 + V4) or between 119 bp-311 bp (ITS1) were chosen for further experiments. The PCR products were mixed in equidensity ratios. Then, the mixture of PCR products was purified with a GeneJET Gel Extraction Kit (Thermo Scientific). Sequencing libraries were generated using a NEB Next^®^ Ultra^™^ DNA Library Prep Kit for Illumina (NEB, USA) following the manufacturer’s recommendations, and index codes were added. The library quality was assessed on a Qubit@ 2.0 fluorometer (Life Technologies, CA, USA) and an Agilent Bioanalyzer 2100 system. Finally, the library was sequenced by the Illumina MiSeq platform at Annoroad Gene Technology Co., Ltd. in Beijing, China.

### Bioinformatic analyses

Quality data files were initially denoised in order to remove sequences that contained sequencing errors. Sequences were removed that had > 2 differences in the primer region, > 1 difference in the barcode, a length of < 200 bp (bacteria) or 150 bp (fungi), or homopolymers that were > 8 nt in length. Unique bacterial sequences were aligned against the Greengenes database (http://greengenes.secondgenome.com/) and trimmed such that only overlapping sequence was considered. Fungal sequences were compared to the recently established unified fungal SILVA database (http://www.arb-silva.de/documentation/background/).

Unique sequences were clustered into operational taxonomic units (OTUs) defined by 97% similarity. A rarefaction analysis was performed. Data preprocessing and OTU-based analyses were performed in Mothur. Principal component analysis (PCA) was performed to explore the differences in bacterial and fungal community structures among all soil samples. To further examine the relationships among the frequencies of the 20 most abundant orders, the samples and the environmental variables, a redundancy analysis (RDA) was performed using CANOCO for Windows [24].

### Statistical analysis

Data were compared using one-way analysis of variance (ANOVA) or two-sample t-test analysis at the end of each bioassay in the IBM SPSS 20.0 software program (SPSS Inc., USA).

### Accession number

The raw bacterial 16S and fungal ITS sequence data were deposited at the National Center for Biotechnology Information (NCBI) under accession number SRP074916.

## Results

### Physicochemical analysis

The initial soil and influence of BIO and chemicial fertiiilizer on the soil physicochemical properties is summarized in Table 3. The pH of the soil decreased with the increase in the BIO concentration. The soil pH in CK, BIO2.5 and BIO5 was no significant change compared with that in the initial soil, but the soil pH in BIO10 and BIO20 was lower than that in the initial soil. The EC, OM, TN, AK and AP of the soil increased with the increase in the BIO concentration. The EC in CK, BIO5, BIO10 and BIO20 was higher than that in the initial soil. The AK and AP in BIO2.5 and BIO5 were lower than those in the initial soil, but these parameters were higher in CK, BIO10 and BIO20 compared to the initial soil. The TN in the initial soil and CK was higher than in all the BIO treatments, whereas the OM in the BIO treatments was higher than that in the initial soil and CK.

**Table 3:** Physicochemical properties of soils.

Treatment	pH	EC (ms/cm)	OM (g/kg)	TN (g/kg)	AK (mg/kg)	AP (mg/kg)
**Initial**	5.72±0.31^a^	0.93±0.05d	44.23±2.1d	1.21±0.06^a^	241.25±17.2c	347.79±17.9d
**CK**	5.51±0.08^a^	1.18±0.05c	44.75±1.2d	1.28±0.01^a^	244.53±15.61c	365.75±25.72c
**BIO2.5**	5.71±0.27^a^	0.9±0.01d	48.38±0.06c	0.55±0.01e	150.85±11.2e	312.34±21.33e
**BIO5**	5.62±0.04^a^	1.12±0.04c	46.82±0.81cd	0.6±0.03d	174.6±10.91d	370.81±30.98c
**BIO10**	5.27±0.02^b^	1.83±0.08^b^	51.17±1.37^b^	0.67±0.01c	295.8±26.03^b^	400.74±31.63^b^
**BIO20**	5.05±0.03^b^	2.37±0.03^a^	53.99±0.71^a^	0.75±0.01^b^	468.65±27.57^a^	457.6±22.63^a^

^a^All values are the mean of three replicates. Numbers followed by “±” represent the standard errors.

^b^Values with different letters after the number in the same column are significantly different at P < 0.05 according to Duncan’s test.

### Disease incidence

**Fig 1:**
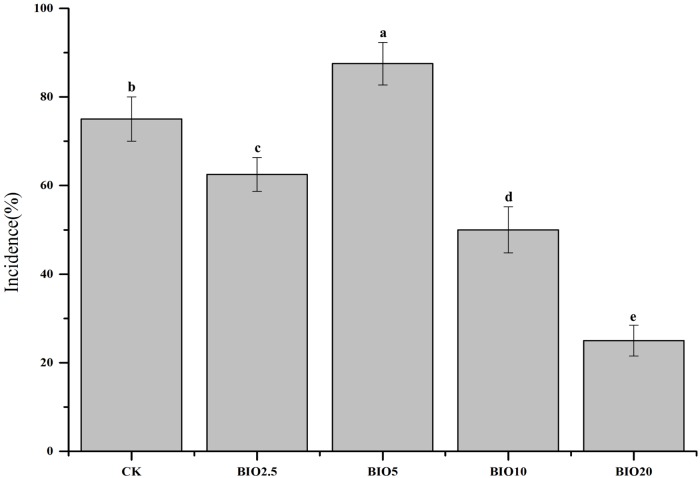
The disease incidence rate of cucumber Fusarium wilt. Columns with different letters are significantly different according to Duncan’s test (*P* < 0.05).

The cucumber disease incidence was significantly different among the five treatments and is show in Fig 1. High incidence of Fusarium wilt occurred in the CK. Compared to the CK, low concentrations of BIO had no obvious inhibitory effect on the disease incidence. The BIO2.5 and BIO5 treatments had an average disease incidence of 62.5% and 87.5%, respectively, and decreased disease incidence was observed in the BIO10 and BIO20 treatments. Disease incidences of 50% and 25% were observed in the BIO10 and BIO20 treatments, respectively.

### Production

**Fig 2:**
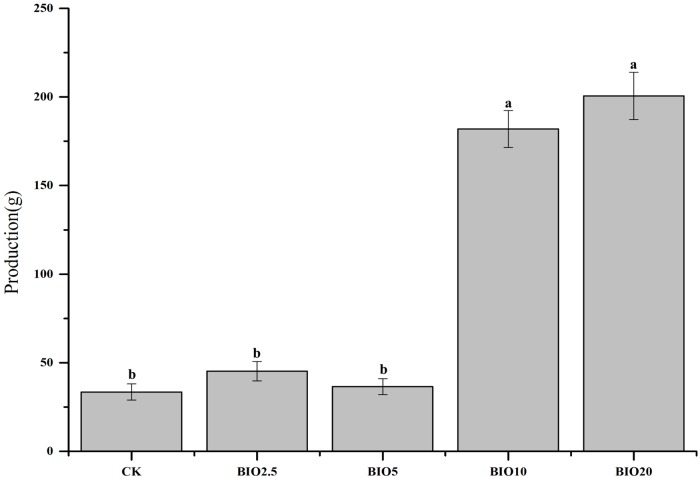
The production of cucumber during 90 days of growth. Columns with different letters are significantly different according to Duncan’s test (*p* < 0.05).

The average production of cucumber under different BIO concentrations is shown in Fig 2. Compared with the CK, no obvious improvement in cucumber production was observed with low BIO concentrations. Significantly increased cucumber production was observed in the BIO10 and BIO20 treatments.

### Relative quantity of *F*. *oxysporum* f. sp. cucumerinum in soil

**Fig 3:**
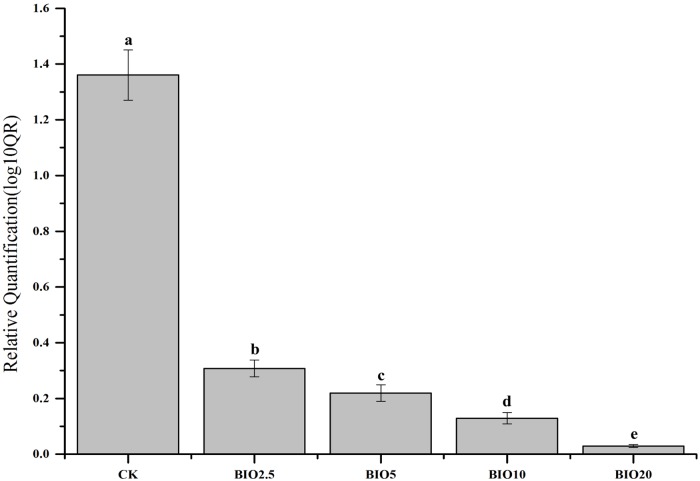
Real-time PCR relative quantification of *F*. *oxysporum* f. sp. cucumerinum in soils. Columns with different letters are significantly different according to Duncan’s test (*p* < 0.05).

Real-time PCR with relative quantitative assays were used to determine the numbers of *F*. *oxysporum* f. sp. cucumerinum in the soil samples (Fig 3). The relative quantity of *F*. *oxysporum* f. sp. cucumerinum in the different treatments were significantly different. Compared to the initial soil, a decrease in *F*. *oxysporum* f. sp. cucumerinum was detected in the all bio-organic fertilizer treatments. However, an increase in *F*. *oxysporum* f. sp. cucumerinum was detected in the CK. The results indicated that BIO can inhibit the growth of *F*. *oxysporum* f. sp. cucumerinum. Compared to BIO2.5 and BIO5, the BIO10 and BIO20 treatments were more effective in reduce of the relative quantity of *F*. *oxysporum* f. sp. cucumerinum, which indicates that higher BIO concentrations could improve the inhibition of *F*. *oxysporum* f. sp. cucumerinum proliferation.

### Relative quantity of *B*. *subtilis* SQR9 in soil

**Fig 4:**
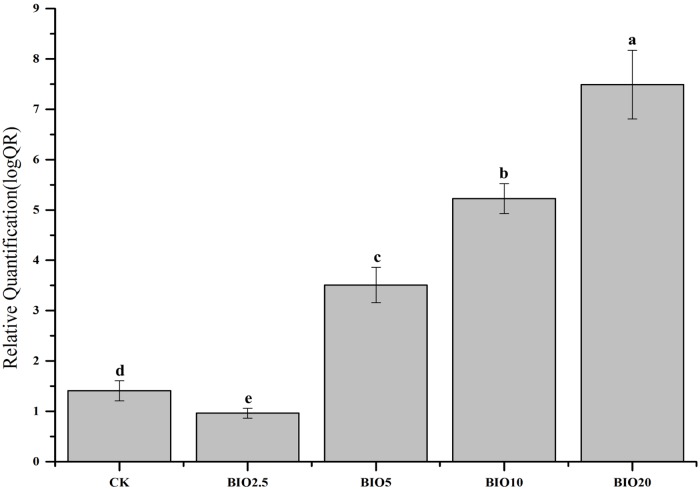
Real-time PCR relative quantification of *B*. *subtilis* SQR 9 in soils. Columns with different letters are significantly different according to Duncan’s test (*p* < 0.05).

Real-time PCR with relative quantitative assays was used to determine the number of *B*.*subtilis* SQR9 in the soil samples (Fig 4). The relative quantity of *B*.*subtilis* SQR9 in the different treatments was significantly different. Compared to the initial soil, the relative quantity of *B*.*subtilis* SQR9 in the CK and BIO2.5 had no obvious increase. However, the *B*.*subtilis* SQR9 in the soil increased with the increase of the BIO concentration. The relative quantity of *B*.*subtilis* SQR9 in BIO20 was approximately seven times than that in the initial soil. The results indicated that higher BIO concentrations could increase the quantity of *B*.*subtilis* SQR9 in the soil.

### CLPPs

**Fig 5:**
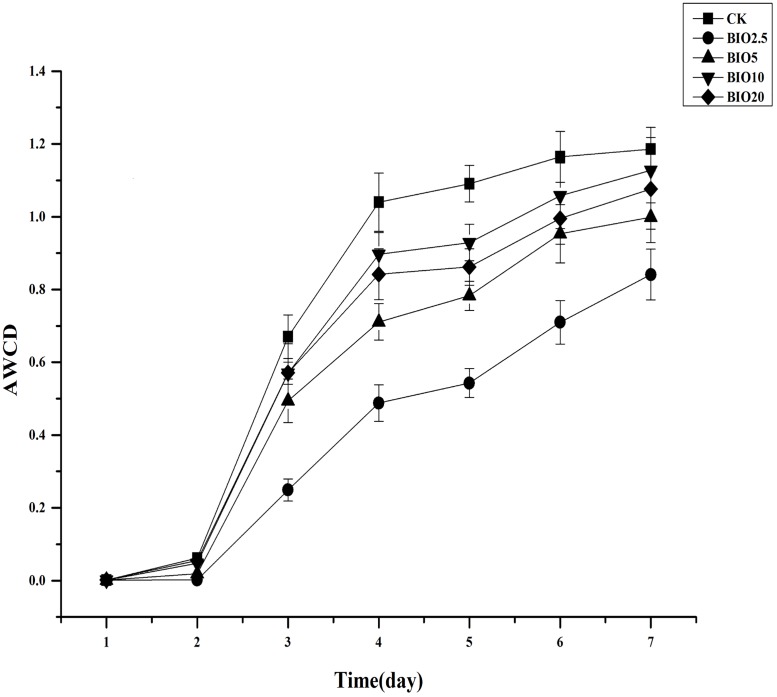
The AWCD for all samples of all carbon substrates on the Biolog EcoPlate over a 7-day incubation at 25°C. The values are the means of three replicate samples.

**Fig 6:**
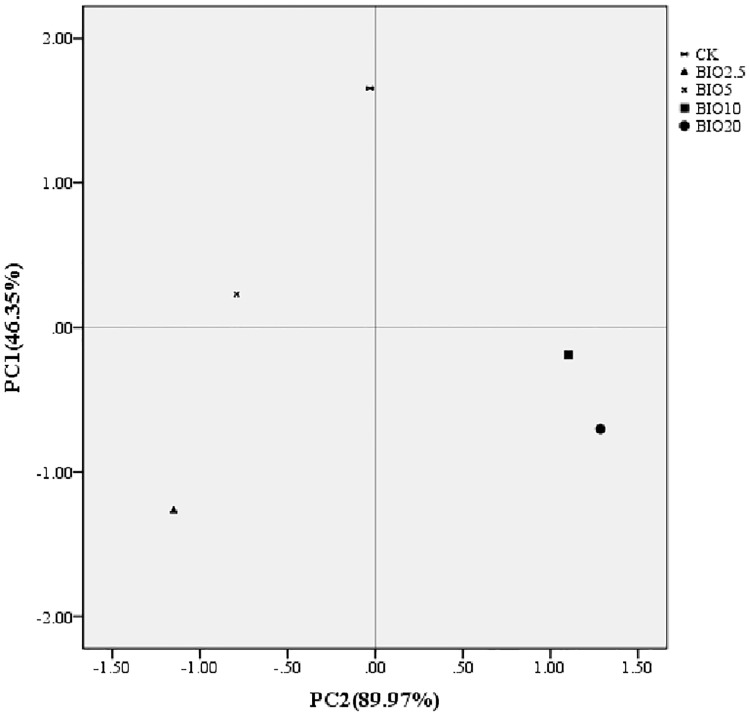
Principal component analysis based on the carbon source utilization pattern of the soil bacterial communities in all samples over a 4-day incubation at 25°C.

The activities of the soil microbial communities were measured with Biolog EcoPlates. The average utilization of C sources (average well color development (AWCD) profiles) increased with the incubation time in all treatments. Overall, soils receiving low BIO concentrations had lower AWCD values than those treated with chemical fertilizer and high BIO concentrations. The AWCD values ranked in the following order: CK>BIO10>BIO20>BIO5>BIO2.5 (Fig 5). The microbial activity in the soil that received a high concentration of BIO was higher than the soil that received a low concentration of BIO. The microbial diversity indices calculated from the CLPP dataset were affected by the different concentrations of BIO applied (Table 4). The CK had higher Shannon and McIntosh diversity indices compared to the other soils, followed by BIO10, BIO20 and BIO5, and the lowest indices were observed for BIO2.5. Principal component analysis was performed for the carbon source utilization pattern. The five soil samples distributed separately at 46.35% and 89.97% on the PCA vector 1 and 2 axes (Fig 6). The soils in BIO10 and BIO20 are closer to each other on these axes, whereas CK, BIO2.5 and BIO5 are very distinct.

**Table 4:** Diversity indices based on the carbon substrate utilization pattern for the soil samples from CK, BIO2.5, BIO5, BIO10 and BIO20.

Diversity measures	CK	BIO2.5	BIO5	BIO10	BIO20
**Shannon diversity index**	6.35±0.12^a^	4.461±0.07d	5.451±0.2c	6.24±0.14^b^	6.311±0.09^a^
**McIntosh diversity index**	3.331±0.07^a^	3.185±0.11c	3.186±0.16c	3.22±0.09^b^	3.301±0.13^a^

^a^All values are the mean of three replicates. Numbers followed by “±” represent the standard errors.

^b^Values with different letters after the number in the same row are significantly different at P < 0.05 according to Duncan’s test.

### Bacterial and fungal community structure

**Fig 7:**
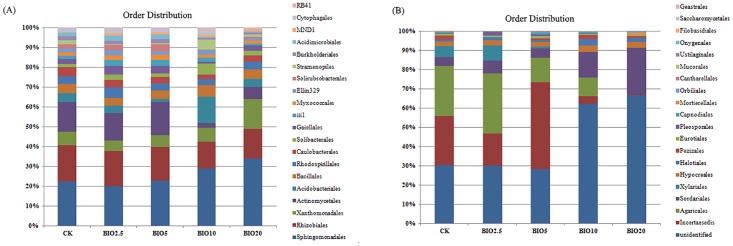
The relative abundances of the dominant bacterial (A) and fungal (B) orders in the five samples.

High-throughput sequencing of 16S rDNA and ITS rDNA genes was used to analyze the bacterial and fungal community structure, respectively, of the CK, BIO2.5, BIO5, BIO10 and BIO20 soil samples. A total of 127514 bacterial sequences were obtained from the five soil samples. The number of bacterial sequences obtained from each sample was 23272, 32093, 23820, 21333 and 26996 for CK, BIO2.5, BIO5, BIO10 and BIO20, respectively (Fig 7A). Among the five samples, these bacterial sequences were mainly classified in followed orders: *Sphingomonadales* (23.2%), *Rhizobiales* (15.1%), *Actinomycetales* (10.1%), *Xanthomonadales* (7.2%), *Acidobacteriales* (4.7%), *Bacillales* (4.2%), *Rhodospirillales* (3.7%), *Caulobacterales* (3.1%), *Gaiellales* (2.7%) and *Solibacterales* (2.5%). *Sphingomonadales* and *Bacillales* were significantly abundant in BIO10 and BIO20 in comparison to the CK, BIO2.5 and BIO5 soils. On the other hand, *Rhizobiales*, *Actinomycetales* and *Caulobacterales* were significantly abundant in CK, BIO2.5 and BIO5 compared to the BIO10 and BIO20 soils. *Xanthomonadales* was significantly abundant in BIO20 in comparison to CK, BIO2.5, BIO5 and BIO10.

The fungal communities in these samples had different relative abundances at the order level. A total of 138896 sequences were obtained from the five soil samples. The numbers of sequences obtained from each sample were 36665, 32602, 31063, 17263 and 21303 for CK, BIO2.5, BIO5, BIO10 and BIO20, respectively (Fig 7B). Among the five samples, these sequences were mainly classified in followed orders: unidentified (39.4%), *incertae sedis* (21.2%), *Agaricales* (18.2%), *Sordariales* (9.2%), *Xylariales* (3.6%), *Hypocreales* (2.7%), *Helotiales* (1.6%), *Pezizales* (1.1%), *Eurotiales* (0.8%) and *Pleosporales* (0.8%). Among the CK, BIO2.5, BIO5, BIO10 and BIO20 soil samples, the unidentified group was abundant in BIO10 and BIO20 in comparison to CK, BIO2.5 and BIO5. *Sordariales* was significantly abundant in BIO10 and BIO20 compared to the other treatments. On the other hand, *incertae sedis*, *Agaricales* and *Xylariales* were significantly abundant in CK, BIO2.5 and BIO5 compared to BIO10 and BIO20 soils.

### OTU-based hierarchical cluster analyses

**Fig 8:**
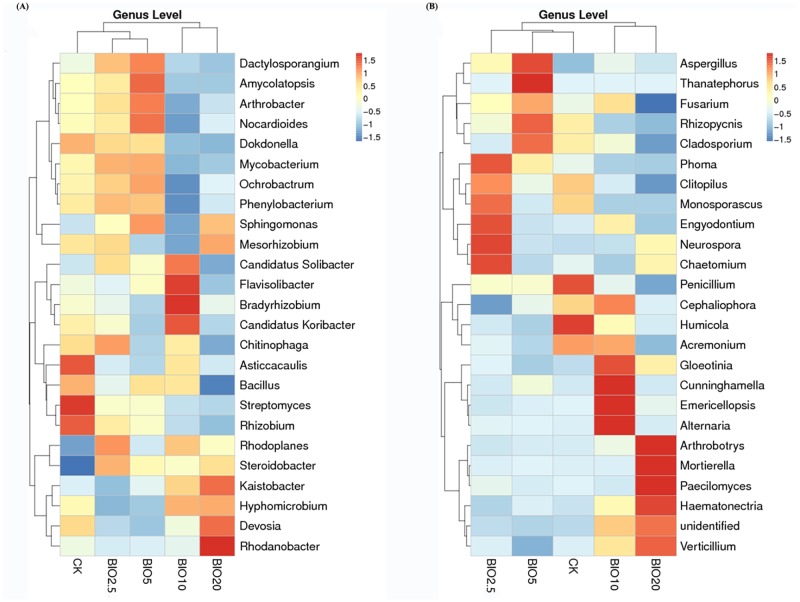
Hierarchical cluster analysis based on the relative abundances of the top OTUs identified in the five samples in the bacterial (A) and fungal (B) datasets.

The heatmap analysis of the top 25 genera with hierarchical clusters based on the Bray—Curtis distance indicated that the community structural patterns were different between the BIO treatments and CK (Fig 8A and 8B). For bacteria and fungi, the five treatments were divided into two broad categories. BIO10 and BIO20 grouped together and were distinct from the other treatments. BIO2.5 and BIO5 grouped together, and this pair then grouped together with the CK. The above results show that BIO2.5 and BIO5 had a certain effect on the soil bacterial and fungal community structure but were not significantly difference from the CK. The difference between BIO10 and BIO20 and the CK was very notable.

### Redundancy analysis

**Fig 9:**
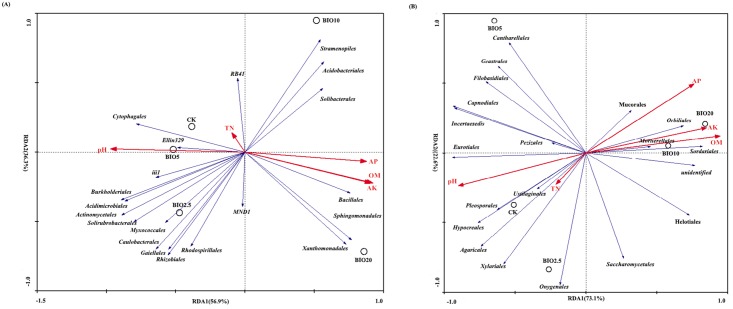
RDA based on the relative abundances of the top 20 bacterial orders and selected soil properties of the soil samples collected from the treatments and CK. Selected soil chemical properties were significantly correlated with variations in the selected bacterial (A) and fungal (B) genera according to the Monte Carlo test (*p* < 0.05). TN: total nitrogen, OM: organic matter, AP: available phosphorus, AK: available potassium.

Redundancy analysis (RDA) was performed to analyze the relationships of the soil microorganisms with the different treatments and soil characteristics. The first and second RDA components explained 93.2% and 95.7% of the total bacterial and fungal variations, respectively (Fig 9A and 9B). For bacteria, the first component (RDA1), which explained 56.9% of the total variation, separated the BIO10 and BIO20 samples from the CK, BIO2.5 and BIO5 samples. As shown by their close groupings and vectors, order of *Xanthomonadales*, *Sphingomonadales*, and *Bacillales* occurred predominantly in the BIO20 and were related to the OM, AK and AP contents, whereas *Cytophagales* occurred predominantly in the CK and BIO5 treatments; *Myxococcales* and *Caulobacterales* occurred predominantly in the BIO2.5. Regarding fungi, the first component (RDA1), which explained 73.1% of the total variation in fungal orders, separated the BIO10 and BIO20 samples from the CK, BIO2.5 and BIO5 samples. As shown by their close grouping and vectors, order of *Orbiliales*, *Sordariales*, and *Mucorales* occurred predominantly in the BIO10 and BIO20 treatments and were related to the OM, AK and AP contents; *Pleosporales*, *Hypocreales*, and *Agaricales* occurred predominantly in the CK; *Onygenales* occurred predominantly in the BIO2.5.

## Discussion

The utilization of antagonistic microorganisms to protect plants from pathogens is a well-documented practice [25,26]. A new biocontrol product, BIO, was prepared by mixing the biocontrol agent *B*. *subtilis* SQR 9 with amino acid fertilizer and matured cow manure. In the present study, the yield and morbidity of cucumber, the relative number of *F*. *oxysporum* f. sp. cucumerinum and *B*. *subtilis* SQR 9 in the soil, and the soil microbial activities and diversity were assessed in the CK, BIO2.5, BIO5, BIO10 and BIO20 treatments.

Different concentrations of BIO had significant impacts on the pH, OM and TN of the soil. The BIO contains acid-hydrolyzed amino acids. The soil pH decreased with the increase in the BIO concentration. Previous studies reported that the soil fertility and plant health increased with an increase of OM content [27]. The OM content of the biological fertilizer was 28.4%, and the amount of OM added to the soil was 3.55 g, 7.1 g, 14.2 g and 28.4 g in BIO2.5, BIO5, BIO10 and BIO20, respectively. The soil OM increased with the increase of BIO concentration. The TN contents of the biological and chemical fertilizers were 2.58% and 15%, respectively. The amount of TN added to the soil was 0.67 g, 0.32 g, 0.65 g, 1.29 g and 2.58 g in CK, BIO2.5, BIO5, BIO10 and BIO20, respectively. The TN increased with the increase in the BIO concentration in the soil but was lower in the BIO applications compared to the CK and initial soil. Reportedly, organic fertilizer can increase the utilization of nitrogen sources by soil microorganisms [28].

The number of pathogenic bacteria and the incidence of cucumber Fusarium wilt have an obvious correlation [25]. The relative number of *F*. *oxysporum* f. sp. cucumerinum of the soil decreased with the increase in the BIO concentration. And the relative number of *B*.*subtilis* SQR9 of the soil increased with the increase in the BIO concentration. The relative number of *F*. *oxysporum* f. sp. cucumerinum and *B*. *subtilis* SQR 9 in the soil revealed that the soils in the BIO10 and BIO20 treatments had the highest *B*. *subtilis* SQR 9 and lowest *F*. *oxysporum* f. sp. cucumerinum (Figs 3 and 4), which agrees with previous results demonstrating that bio-organic fertilizer treated soil can increase the abundance of *B*. *subtilis* SQR 9 and decrease the abundance of *F*. *oxysporum* f. sp. cucumerinum [29]. The colonization of *B*. *subtilis* SQR 9 along cucumber roots could suppress the amount of *F*. *oxysporum* f. sp. cucumerinum and reduce the incidence of Fusarium wilt [7]. Applying high concentrations of biological fertilizer to the soil significantly reduce the disease incidence and improve the crop production (Figs 2 and 3).

Bacteria were the main constituents of the soil biomass, accounting for 70% to 90% of the total soil microorganisms [30]. Fungi are important in the process of decay, which can break down cellulose, lignin and pectin and enhance soil fertility [31]. Most of the microbial species in the soil were positively correlated with the nutrient content in the soil and crop yield. CLPPs have been wildly used to study microbial activity [32] and were used to demonstrate the effect of BIO on the soil microorganisms in the present study. The application of organic fertilizer at different concentrations has a series of effects on soil microbial proliferation and activity [33]. The Biolog-based functional Shannon diversity and McIntosh diversity indices showed a collapse of microbial functional diversity in BIO2.5 and BIO5 soil compared to the other treatments, which means that a low concentration of BIO applied to the soil did not increase the soil microbial activity. Therefore, the soil microbial activity varied with the varying nutrient concentration in the soil. A high concentration of BIO applied to the soil significantly increased the soil microbial activity compared with BIO2.5 and BIO5. Principal component analysis based on the carbon source utilization pattern of the CK, BIO2.5, BIO5, BIO10 and BIO20 soil samples clearly showed that the functional diversity in BIO10 and BIO20 was more similar compared to the other treatments. The functional diversity in BIO2.5 and BIO5 changed compared to the CK, but no similarity was observed. Our CLPP-based PCA suggests that the change in activity and functional diversity of the soil microbial community was related to an altered concentration of bio-organic fertilizer, which in accordance with the high-throughput sequencing results.

In the present study, 16S rRNA and ITS rRNA gene-based approaches (high-throughput sequencing) were used to study the microbial communities. Based on the hierarchical cluster analysis results, the soil bacterial and fungal community structures observed in the BIO10 and BIO20 treatments differed from those in the other treatments, similar to previous findings in which different concentrations of organic fertilizer altered the soil microbial community structures [34]. Bio-organic fertilizer was an effective approach to suppress the Fusarium wilt of cucumber through recovering the microbial populations damaged by *F*.*oxysporum* f. sp. cucumerinum [29]. At order level, *Sphingomonadales*, *Bacillales*, *Solibacterales* and *Acidobacteriales* did not increase in BIO2.5 and BIO5 compared with the CK but increased significantly in BIO10 and BIO20. Among these orders, *Sphingomonadales* accounted for the greatest numbers in the soil. Some reports have stated that *Sphingomonadales* are photosynthetic bacteria and play a very important role in the synthesis of sugars, amino acids, vitamins and other bioactive substances. *Bacillales* and *Solibacterales* are expected to result in enhanced cycling of essential micro- or macronutrients, which may partially improve the soil fertility and plant growth efficiency [35,36]. *Acidobacteriales* can dissolve lignin and cellulose, providing nutrients for plants [37]. Similar to the bacterial communities, BIO significantly affected the abundance and composition of fungal communities. *Sordariales* exists in animal feces, and their numbers increased with the increase in the BIO concentration in the soil [38]. *Xylariales* are particularly significant filamentous pathogenic fungi that can cause crop root rot [39]. BIO10 significantly decreased the amount of *Xylariales* in the soil compared with CK, BIO2.5 and BIO5, and this order was not detected in BIO20.

RDA analysis revealed that *Xanthomonadales*, *Sphingomonadales*, *Bacillales*, *Orbiliales*, *Sordariales*, and *Mucorales* occurred predominantly in the BIO10 and BIO20, which were related to the OM, AK and AP contents. *Cytophagales*, *Pleosporales*, *Hypocreales*, and *Agaricales* occurred predominantly in the CK, which were related to pH and TN. Bio-organic fertilizer increased organic matter, available P and available K contents in the soil, which were claimed to be positively association with the suppression of *Fusarium* spp. for different crops [40,41].

## Conclusions

To summarize, our study demonstrated that the suppression of Fusarium wilt in cucumber by BIO increases with increasing BIO concentration, which increases cucumber production. A high concentrations of BIO reduced the *F*. *oxysporum* f. sp. cucumerinum population, increased the *B*. *subtilis* SQR 9 population and soil fertilizer, altered the microbial structure and stimulated potentially beneficial microbial consortia, i.e., *Sphingomonadales*, *Bacillales*, *Solibacterales* and *Sordariales*.
